# Cortical computations via transient attractors

**DOI:** 10.1371/journal.pone.0188562

**Published:** 2017-12-07

**Authors:** Oliver L. C. Rourke, Daniel A. Butts

**Affiliations:** 1 Program in Applied Mathematics, Statistics and Scientific Computation, University of Maryland, College Park, MD, United States of America; 2 Department of Biology and Program in Neuroscience and Cognitive Science, University of Maryland, College Park, MD, United States of America; University of Chicago, UNITED STATES

## Abstract

The ability of sensory networks to transiently store information on the scale of seconds can confer many advantages in processing time-varying stimuli. How a network could store information on such intermediate time scales, between typical neurophysiological time scales and those of long-term memory, is typically attributed to persistent neural activity. An alternative mechanism which might allow for such information storage is through temporary modifications to the neural connectivity which decay on the same second-long time scale as the underlying memories. Earlier work that has explored this method has done so by emphasizing one attractor from a limited, pre-defined set. Here, we describe an alternative, a Transient Attractor network, which can learn any pattern presented to it, store several simultaneously, and robustly recall them on demand using targeted probes in a manner reminiscent of Hopfield networks. We hypothesize that such functionality could be usefully embedded within sensory cortex, and allow for a flexibly-gated short-term memory, as well as conferring the ability of the network to perform automatic de-noising, and separation of input signals into distinct perceptual objects. We demonstrate that the stored information can be refreshed to extend storage time, is not sensitive to noise in the system, and can be turned on or off by simple neuromodulation. The diverse capabilities of transient attractors, as well as their resemblance to many features observed in sensory cortex, suggest the possibility that their actions might underlie neural processing in many sensory areas.

## Introduction

The real world “causes” of sensory inputs usually persist for much longer than the time scales of neural processing in sensory areas. As a result, there is great utility for neural and circuit mechanisms within sensory cortex that can hold information for several seconds, much longer than the timescale of neural integration. Storage of information on this time scale is commonly addressed in the context of “short-term memory” [1], but there is more general utility for seconds-long storage of information. For example, such aggregation of information over time can be used to segregate auditory stimuli into perceptual auditory objects [2]. Similarly, features of visual objects can be assembled over time using such associations despite temporary occlusions and visual noise [3].

The most common models of short-term memory rely on the concept of a “persistent attractor” [4,5]. A network with a fixed set of recurrent connections can support “attractors”, which correspond to particular patterns of activity that remain stable or decay slowly with seconds-long time scales. In this context, placing the network in one of these attractors (via inputs) can result in short-term memory, which can be ‘recalled’ by observing the activity at a later time (before the attractor decays). Persistent activity is typically maintained by a combination of excitatory and inhibitory activity [6,7], and persistent states can even exist in random networks with particular properties [8]. The unifying feature of persistent attractor networks is that information is stored in neural activity itself, thus keeping it readily accessible.

The persistence of memory-specific neural activity in certain cortical regions during short-term memory tasks has been cited as evidence supporting the persistent attractor hypothesis for short-term memory [9,10]. More recently, however, it has been shown that this activity is not necessary for the persistence of the underlying memories [5,11,12], and that some form of short-term memory also occurs in the sensory cortices themselves [13–15]. An alternative location for the storage of information about recent inputs is in the local connectivity within the network itself. Indeed, such memory storage is implicit in models of long-term memory [16], where memories are encoded in the excitatory connectivity which is established using a simple form of associative plasticity. Such a scheme could also be used for short-term memory if such changes in synaptic connectivity were temporary, allowing for the short-term preservation of information within the network without affecting the network’s long-term structure [17–20]. The temporary change would support a particular attractor in the presence of appropriate inputs [21], thus allowing for memory recall over this period. We label such attractors ‘transient’ as they only exist during appropriate input and due to relevant changes to network connectivity (which are themselves temporary).

Here, we propose transient attractors as a unifying mechanism within cortical networks that can support multiple types of computation that require combining information across time scales longer than those of the underlying neurons (similar to another recently published model [22]). We first demonstrate how a transient attractor functions in the context of a classic short-term memory task. Several memories can be stored in the network structure, allowing for their recall in the presence of suitable inputs. These memories then fade over several seconds. The same network can be used to extract information from time varying stimuli, specifically in the tasks of stream segregation and signal de-noising. We finish by considering some issues that impact the various uses of transient attractors, including transient attractor maintenance, the effect of top-down attention and the overall robustness of the network.

## Results

**Fig 1:**
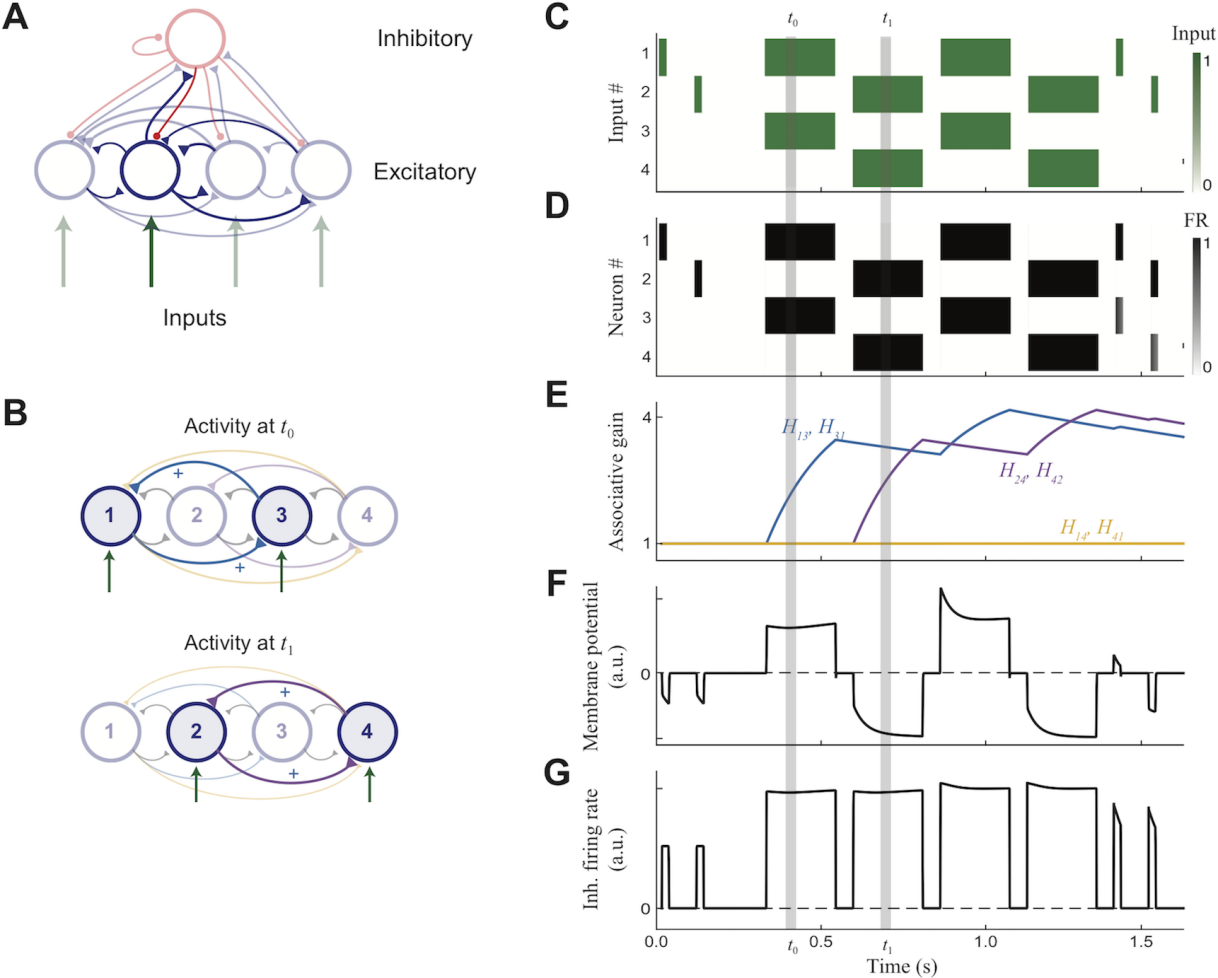
Transient attractors in single layer network via associative weight modifications. (A) Network structure. (B) When presented with stimulus, recurrent connections between simultaneously active neurons are strengthened. (C) Stimulus: two patterns shown successively at 4 Hz, capped at beginning and end by probe (D) Activity of excitatory neurons in response to stimulus (E) Weight changes for representative sample of recurrent connections. (F) Potential of sample excitatory neuron #3. Initially, both probes cause some inhibition while after training the in-pattern probe causes elevated potential (firing), while other probe causes increased inhibition. (G) Inhibitory cell’s firing rate.

We consider here a simple form of a transient attractor network (Fig 1A), which demonstrates the basic behavior without requiring intricate models of any one process. To this end, each neuron’s activity is summarized by a single, continuous variable, the firing rate (*y*_*i*_ (*t*) for neuron *i* at time *t*). This is calculated using a standard firing rate model (see Methods) that integrates recurrent excitation and inhibition, along with feedforward inputs which represent the stimulus. Short-term memory is supported within the network by varying the recurrent excitatory currents.

The network behavior is then shaped primarily by the dynamics of recurrent excitation. At any given moment, the strength of a recurrent excitatory connection between (postsynaptic) neuron *i* from a (presynaptic) excitatory neuron *j*, *W*_*ij*_(*t*), is the product of three terms: a fixed baseline synaptic weight *S*_*ij*_, an associative (Hebbian) gain *H*_*ij*_(*t*), and a synaptic depression term *x*_*i*_(*t*):

$$W_{ij}(t)=S_{ij}H_{ij}(t)x_{i}(t)$$ (1)

The Hebbian plasticity term *H*_*ij*_(*t*) increases with coincident pre- and postsynaptic activity *y*_*i*_ (*t*)*y*_*j*_(*t*), and decays towards some minimum value *H*_*min*_ in the absence of any coincident activity:

$$\frac{{dH}_{ij}(t)}{dt}=\frac{1}{\tau_{H_{+}}}[H_{max}-H_{ij}(t)]y_{i}(t)y_{j}(t)-\frac{1}{\tau_{H_{-}}}[H_{ij}(t)-H_{min}]$$ (2)

The growth term is scaled so that the connection strength cannot exceed a maximum value *H*_*max*_. The rates of growth and decay are governed by their respective timescales, $\tau_{H_{+}}$ and $\tau_{H_{-}}$ (with rate of growth significantly faster than that of decay).

Excitation is regulated by (and stable due to) two mechanisms: feedback inhibition, and the synaptic depression term *x*_*i*_(*t*). For this simple network, we only consider a single inhibitory unit, which receives inputs from, and projects back to, the excitatory neurons and itself; connections to and from the inhibitory neuron are uniform. This inhibitory unit therefore suppresses all neurons by an amount proportional to the total excitatory activity, resulting in competition between the excitatory neurons. Synaptic depression *x*_*i*_(*t*) is governed by a standard model [23]:

$$\frac{dx_{i}(t)}{dt}=\frac{1}{\tau_{x_{+}}}[1-x_{i}(t)]-\frac{1}{\tau_{x_{-}}}[x_{i}(t)y_{i}(t)]$$ (3)

This decreases the strength of a given connection *W*_*ij*_(*t*) (Eq 6) due to presynaptic activity *y*_*i*_(*t*), and otherwise increases back to a baseline (unity).

In this simple network, the baseline strength is assumed to be uniform (*S*_*ij*_ = *S*_0_). As we will describe, this gives the network the maximum potential for memory storage, but alternatives will be considered later.

### Short-term memory via transient attractors

The behavior of this network can be understood in the context of attractor dynamics [24]. In the presence of a constant external input, firing rates in the network will settle into a stable pattern of neural activity–an attractor–that depends on both the external input and the state of the network. Note that such a definition of an attractor is broader than that used in much of the persistent attractor literature, which only considers attractors that remain active when external input is removed. Because both the stimulus and effective synaptic strengths can change in time, the attractor for a given network itself is time-varying, and–crucially–will depend on recent history of network activity through the associative gain term (*H*_*ij*_). This approach of the memory being the attractor that results from time-varying synaptic strengths–and not the neural activity itself–not only allows for more flexible storage of information, but also the targeted recall of certain memories and effects a significant reduction in the interference between simultaneously stored memories.

We first illustrate how the transient attractor network works within a minimal network with just four excitatory neurons (Fig 1A). We select two patterns to store: the first with neurons #1 and #3 coactive, and the second with neurons #2 and #4 coactive (Fig 1B). Before the memory is stored, we present “probe” stimuli, each driving a single neuron (Fig 1C, *left*) in order to verify there are no preexisting network attractors. Indeed, such probe stimuli only evoke activity in the neurons that were externally stimulated (Fig 1D, *left*). To imprint the memory, the two patterns are displayed alternately at 4 Hz for 1 sec (Fig 1C, *center*). Following this, both probe stimuli are displayed again (Fig 1C, *right*) to determine if the memories are recalled in the network activity. Indeed, while only the stimulated neurons fire in response to the probe stimuli at the beginning (Fig 1D, *left*), the patterns emerge after training (*right*).

During the training period, the memory is imprinted in the increased recurrent weights between coactive neurons over repeated presentations (Fig 1E). These strengthened connections then lead to increases of membrane voltages when even a part of the recently imprinted pattern is shown (Fig 1F). This in turn causes an increase in inhibitory firing rates proportional to the additional excitatory activity (Fig 1G), and an increase in suppression of the non-paired neurons.

**Fig 2:**
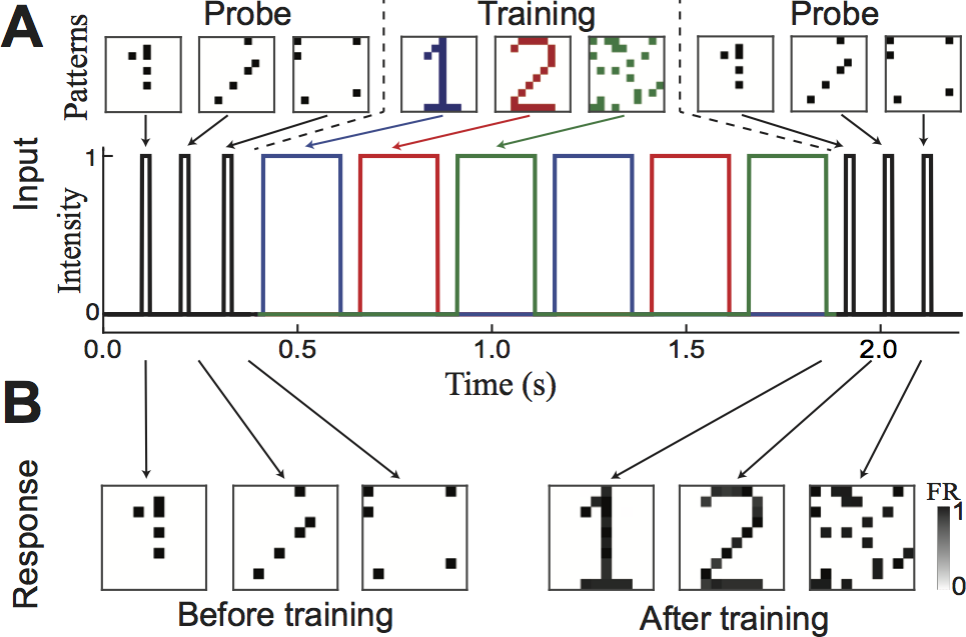
Transient attractors store several arbitrary patterns. (A) Stimulus composed of probe stimuli and training stimuli, with three different patterns (two recognizable patterns, one random, all overlapping). Probes are random subset of 25% of each pattern respectively. (B) Excitatory activity (firing rate) at time of probes.

We next extend this simple example to a much larger network, capable of learning multiple, overlapping patterns. This network has 100 excitatory neurons, arranged in a 10×10 grid. Note that the grid arrangement is only to make visualizing the patterns of activity easier, and it does not represent any biases in connectivity; the excitatory connections are all-to-all, and of equal strength. We train this network with three patterns, two digits (to be easily recognizable) and a third composed of randomly selected neurons. This set of patterns illustrates how any pattern can be stored in the network, but also note that the two digits chosen have a large number of shared elements. Random subsets of each pattern are selected as probe stimuli, and the network is tested to have no preexisting attractors, and trained as described above (Fig 2A). The successful storage of the memories in the network can be verified by comparing the levels of activity of the excitatory neurons to the initial and final probes (Fig 2B). This shows that an attractor has been created for each pattern. Furthermore, due to the inhibition-mediated competition, activity does not ‘leak’ between overlapping attractors, and the stored information is recalled in the presence of a relevant probe. This demonstrates that this network is capable of performing short-term memory tasks involving multiple (potentially overlapping) memories held simultaneously. As with Hopfield networks, the memory capacity of this network (i.e., the number of patterns that can be stored simultaneously in memory) increases with the number of neurons [25], but in practice such a capacity cannot realistically be used due to the limitation of the transient time scale over which the trained patterns of connectivity maintain themselves.

**Fig 3:**
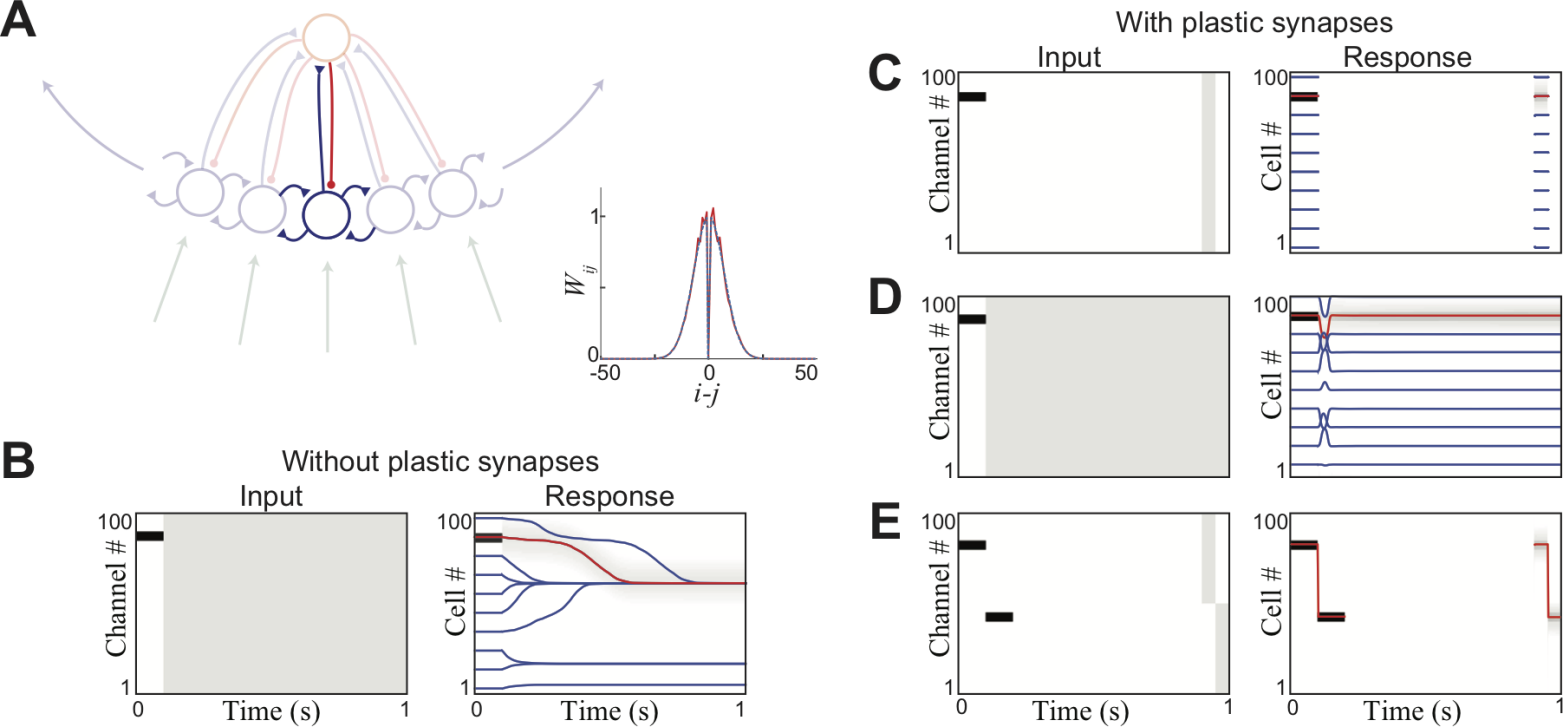
Short-term memory in a ring attractor. (A) Structure of ring attractor (inset: bidirectional excitatory weight from neuron i to neuron j). All plots have noise (epsilon) = 0.05. (B) Persistent activity subject to drift. Center of distribution of activity shown for ten initializations, one typical trajectory shown by shading. (C) Plastic synapses allow for information storage in transient attractors at any single location (D) Transient attractors stabilize activity in case of persistent activity (initial departure due to immediate depressive feedback) (E) Transient attractors allow for simultaneous storage of multiple locations (recall prompted by stimulating either upper or lower half of cells).

Stored short-term memories in this network have an additional attractive property in contrast to persistent-activity-based attractors: namely that they are stable while being stored. Such stability can be demonstrated in an example network where there is a clear topography between different activity states of the network. Thus, we next consider a ring attractor [26]. A ring attractor is composed of a circle of neurons, with each neuron preferentially connected to its neighbors (Fig 3A). In principle, ring attractors based on persistent activity can store a continuous variable because activity at any point on the ring can be stable. However, it has been shown that any noise in recurrent connections will cause a severe reduction in the number of stable equilibriums: typically down to a handful [27]. In practice, this means that the system will always drift to one of the relatively few global attractors (Fig 3B).

Transient attractors avoid this drift by having the network inactive in between training and read-out (Fig 3C), meaning that the memory cannot drift. Any unpatterned noise in the intervening period will not consistently activate pairs, and thus the presence of the attractor itself will also be robust to noise (see below). This observation complements earlier work [27] showing plastic synapses will reduce the rate of drift in the case of persistent activity (Fig 3D). Furthermore, analogous to the more general network considered above (Fig 2), this network is capable of storing multiple locations simultaneously (Fig 3E), each re-activated by their own probe. This demonstrates how storing information in modified synaptic connections, as opposed to persistent activity, prevents slow distortion of the information by small errors within the network (in this case, attractor drift).

### Maintenance of information over time

By design, information stored in transient attractors degrades at the time scale of the underlying transient synaptic plasticity. While this would appear to limit the amount of time a memory can be stored by the transient attractor, such a network can extend to storage over longer periods of time through reactivation of the attractor [18]. Such reactivation will strengthen all relevant connections, and thereby allow information to be stored for durations well past the time scales of the decay of the transient synaptic plasticity.

**Fig 4:**
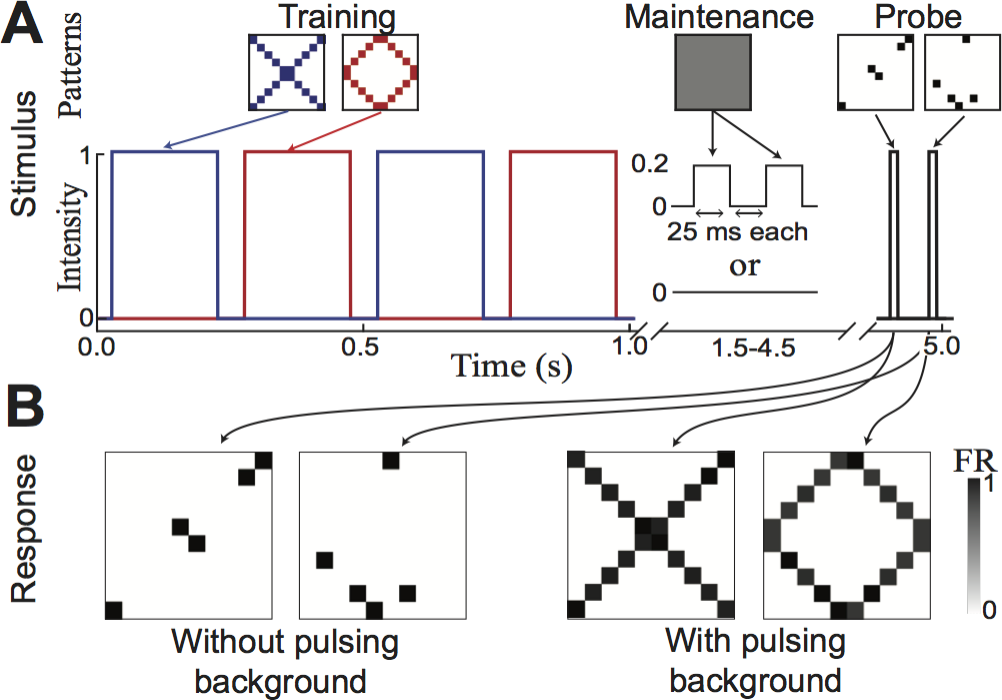
Network can separate patterns using temporal coherence. (A) Two training patterns and their temporal envelopes. (B) Excitatory activation at time of probes revealing transient attractors have formed for each pattern.

To demonstrate how the transient attractor is capable of this, we first store two overlapping patterns (Fig 4A, *left*). Without any further activity, the information stored will become inaccessible over several seconds due to the timescale of decay of the induced synaptic plasticity. However, here the stored information is refreshed by regular reactivation of the attractors via pulsing background activity (Fig 4A, *center*). Background stimulation causing the refresh need not be specific to any stored pattern; in this example, background stimulation is uniform across all channels, but as a result momentarily activates individual attractors within the network. Furthermore, the pulsing nature allows for sequential activation of multiple attractors due to the synaptic depression of synapses which were most recently activated. The pulsing uniform activity is not the only conceivable method of refreshing memories; for example, specific memories might be targeted using an appropriate probe. As a result of this attractor reactivation, it can be seen that the duration of the memories has been extended (Fig 4A, *right* and Fig 4B). This demonstrates the how transient attractors could store information over variable time scales.

### Associating distinct patterns of input via temporal coherence

For the above examples of memory, stimuli were presented separately in time in order to focus on the storage and retrieval of patterns. However, real world stimuli will often not be so conveniently separated in time, with different components that can only be distinguished by detecting shared temporal features. Such a theory of “temporal coherence” has been suggested as a solution for the “cocktail party” problem, that is the ability to associate the features comprising different sounds and focus on those components while suppressing others [28,29]. Temporal coherence has likewise been used for visual object separation [3].

**Fig 5:**
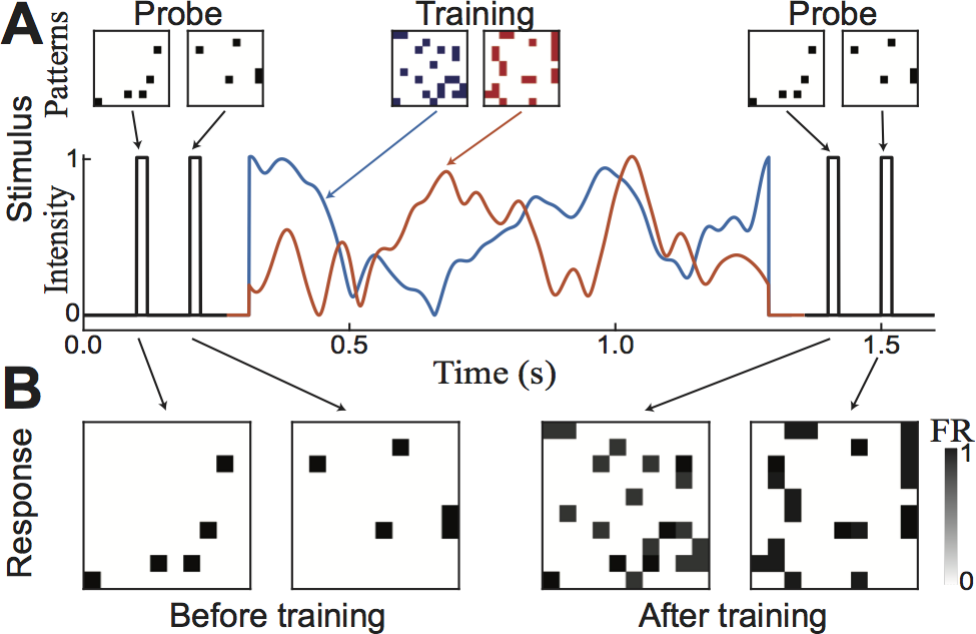
Transient attractors for de-noising and object recognition. (A) Stimulus composed of two parts. Signal (top) is occluded pattern (25% occlusion) for 25 ms, repeats every 100ms. Noise (bottom) random across all non-signal channels. Noise and signal have approximately same amplitude, average activity and temporal correlations. (B) Network activity in response to stimulus. Initially network responds to noise and signal equally, but over time correlations in input allow it to filter out noise and complete the pattern.

The network described above can perform a simple example of such segregation based on temporal coherence. The training stimulus is composed of two random, non-overlapping patterns of activation, which are then modulated by two random and independent temporal envelopes (Fig 5A). As with earlier examples, probes are displayed before and after exposure to patterns to demonstrate the creation of transient attractors. While both patterns were present at some amplitude throughout the training period, the network responses to the probes (Fig 5B) following training reveal that the network has learned both patterns. This happens due to the inhibitory feedback which prevents both patterns from being represented simultaneously. As patterns in the network are not represented simultaneously (even if both are present in the stimulus), they are essentially temporally segregated within the network allowing associations to be learned. Conversely, any inputs which have been co-active for a significant period of time are temporally associated, and will be bound while the two inputs are displayed. We conclude that the network is capable in-principle of performing some form of on-line temporal coherence analysis [30].

### Separating signal from noise

**Fig 6:**
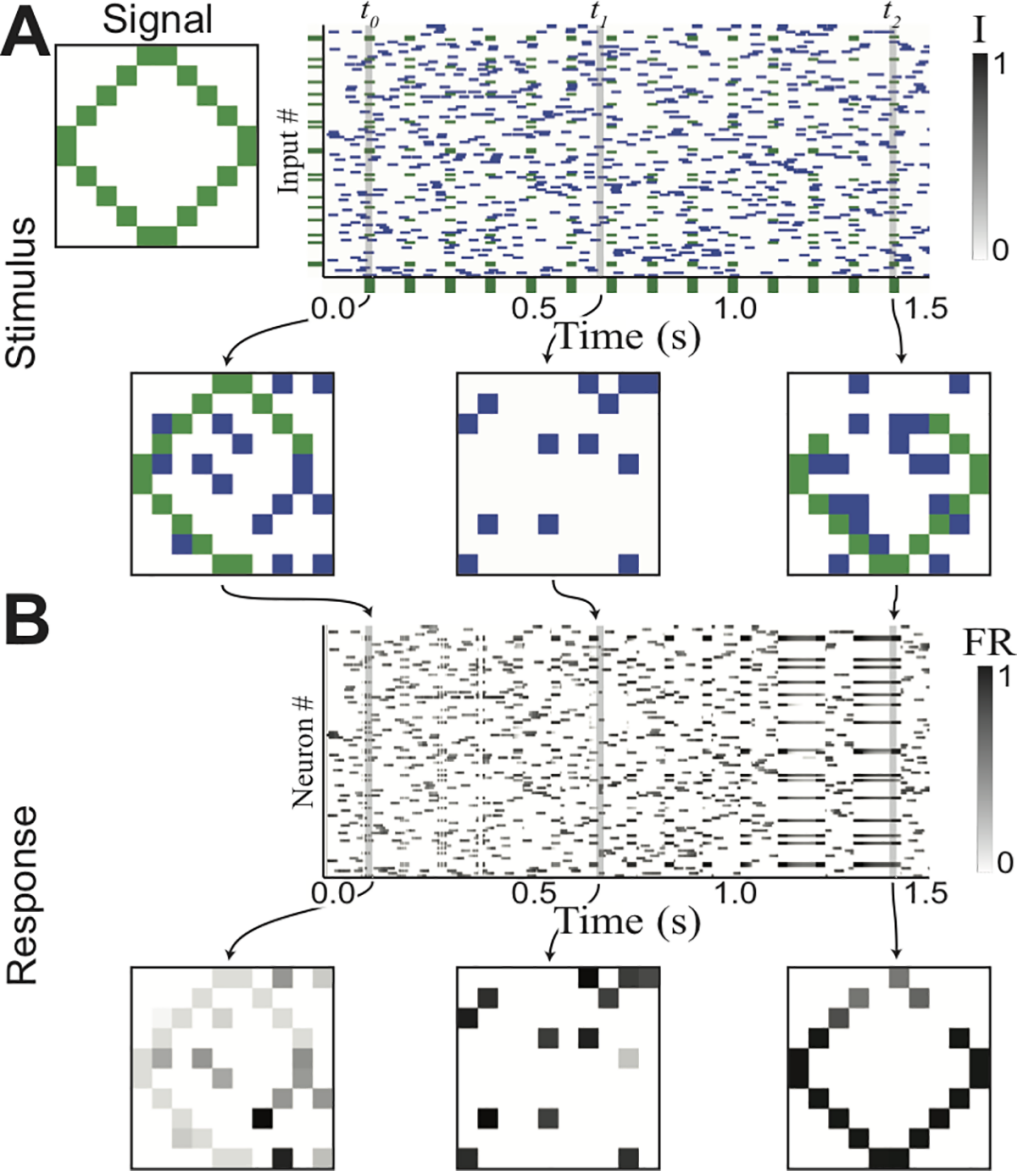
Maintenance of transient attractor by uniform input. (A) Two overlapping patterns stored in memory during first second, recall attempted between 4.8 and 5 seconds. Intermediate period filled with either pulsing low intensity uniform network inputs (top) or no input (bottom). (B) Continued activity allows network to maintain transient attractors and extends duration of memory.

Just as networks with persistent activity may act as neural integrators [31], the transient attractor network may also act as an integrator, allowing it to filter out noise and store an uncorrupted version of the signal. This works because changes to network connectivity sum for short time scales (those less than the time scale of decay). We demonstrate this ability with an example where the signal corruption is due to both occlusion (part of pattern temporarily absent) and uniform noise (additional spurious inputs). We construct a stimulus composed of two parts, signal and noise (Fig 6A). Different partially occluded versions of the pattern are presented briefly. Noise is also introduced, with other inputs randomly active such that the average firing rate is constant across all inputs.

In the context of such stimulation, it is not possible to distinguish between signal and noise by examining either any individual channel over all time, or all channels together at one individual point in time. However, because the plasticity integrates over all temporal associations on the second-long time scale, the noise ends up contributing much less to the connectivity compared with the more consistent signal over this time scale, resulting in an attractor dominated by the combinations of associates that got presented. By the end of training, presentation of a part of the pattern will activate a transient attractor corresponding to the entire pattern (Fig 6B), both filtering out the noise and filling in the majority of the occluded channels.

### Modeling attention and the role of inhibition

**Fig 7:**
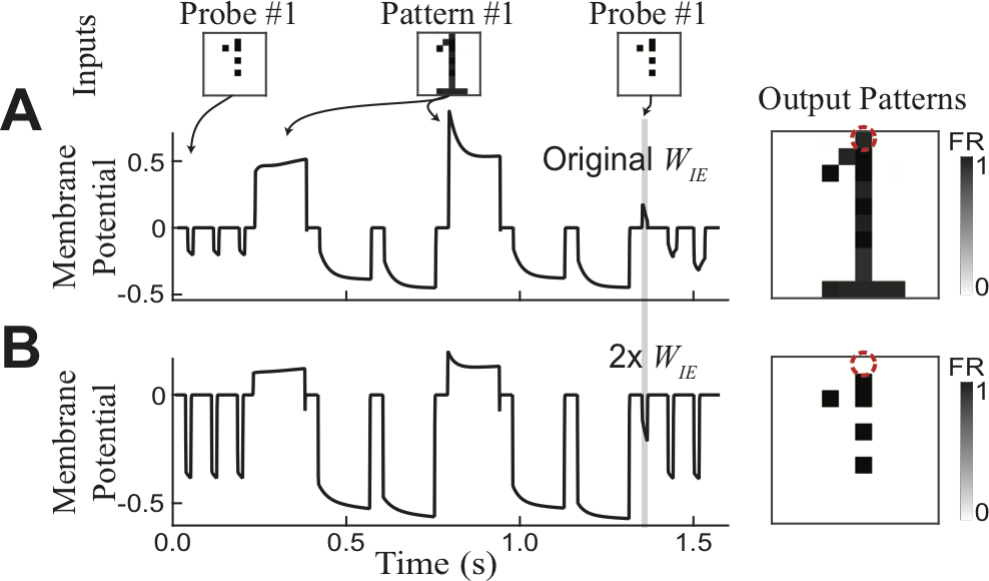
Inhibition as proxy for attention. Network from Fig 1A with either standard (A, C) or increased (B, D) levels of inhibition. Excitatory neuron responses (A, B) and potentials (C, D) reveal dependence on level of inhibition, and suggest inhibition as proxy for attention.

The transient attractor network also has the ability to turn on or off its function through straightforward modulation of inhibition. When the overall strength of inhibition is increased, recurrent activation of attractors will be suppressed such that the network will have no attractors other than faithfully relaying the stimulus. To demonstrate this, we consider the network described in Fig 2, and re-run the simulations when the level of inhibition is increased by doubling the strength of all inhibitory synapses. Although exposure to patterns still leads to synaptic strengthening, such changes are insufficient to create a stable attractor, and the final probe no longer leads to pattern recall (Fig 7). In this example, inhibitory modulation works to prevent retrieval of previous associations. Such basic modulation coincides with observations of the requirement of attention or engagement for the storage of short-term memories [9], as well as for changes associated with auditory streaming [29], and is generally useful to selectively perform the various functions of a transient attractor network.

### Model robustness

**Fig 8:**
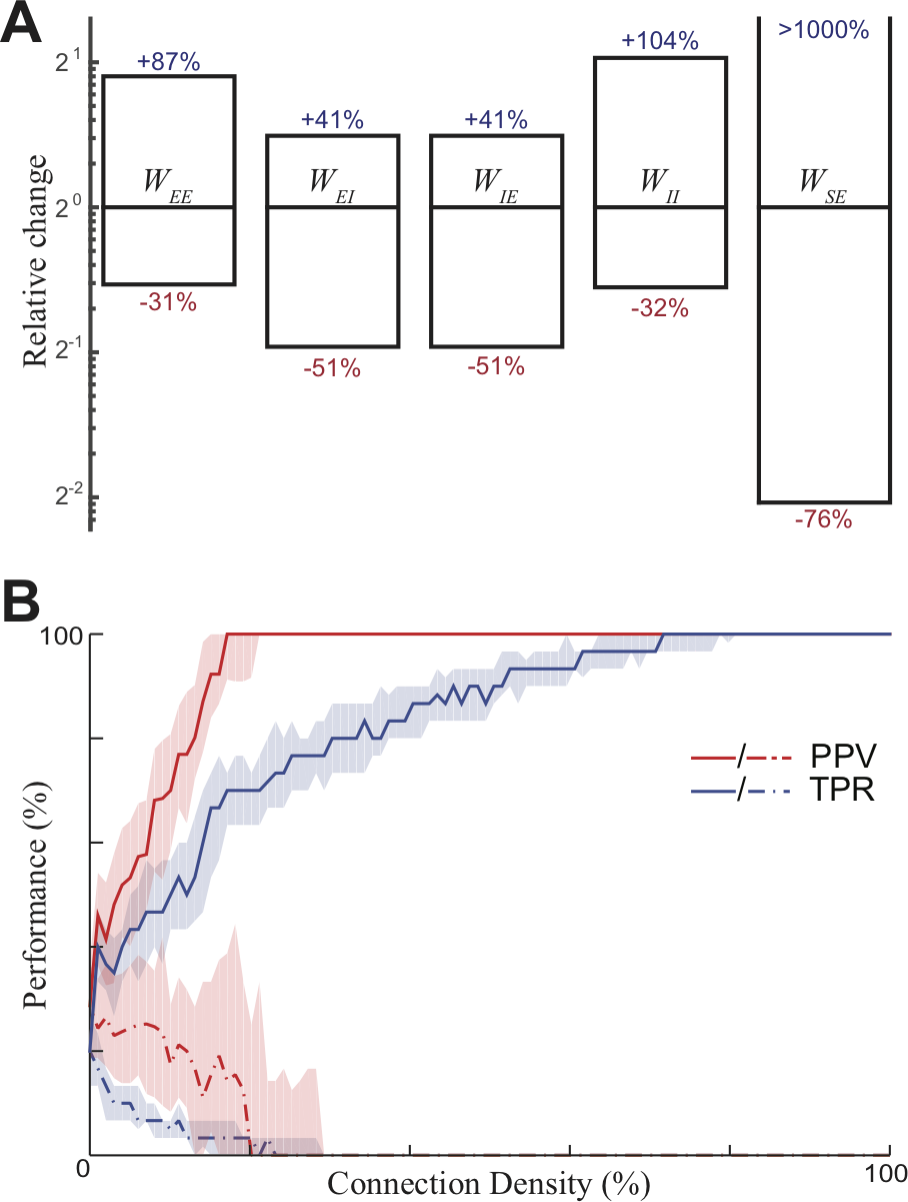
Network resilience. (A) The network continues to be able to successfully recall information for a wide variety of values of each parameter (ratio compared to default plotted). (B) Network performance for sparse network over 100 trials. PPV = Positive Predictive Value, TPR = True Positive Rate. Dashed/solid lines are median before/after training, shaded region lies between first and third quartile.

Stability is often a large concern in neural networks with recurrent excitation; a slight modification to the strength of recurrent connections can either lead to runaway excitation or silence activity throughout the network. We can test how fine this balance is in our model by changing the baseline synaptic strengths of all neurons of a certain type, for example halving all feedback inhibition, and determining if the network continues to successfully store and recall patterns. Each individual parameter could be varied by at least 25% in either direction (Fig 8A), showing the model to be highly resilient to the average sizes of synaptic strengths. We attribute this stability to the close link between inhibition and excitation, as the amount of inhibition scales with the amount of excitation, similar to many E-I networks [24]. Additional stability to the network is a result of saturating firing rates within the single-neuron models.

We also perform a much more extreme manipulation. We randomly removed a percentage of recurrent connections while keeping total recurrent connection strength constant. Such a manipulation results renders the network structure highly heterogeneous. It was found that the network still functions remarkably well at recalling any pattern for connection densities as low as 20% (Fig 8B). This result comes from the manner in which memories are stored–as associations between many different pairs of neurons–which is only perturbed when a large proportion of connections have been removed. This demonstrates that the underlying functionality of the network is not overly reliant on a homogeneous network structure, and therefore may function well within biological networks that can be highly heterogeneous in nature.

The transient attractor model becomes more robust in larger networks; the larger number of neurons comprising each pattern make it exponentially less likely that any two patterns will significantly overlap (relative to the number of neurons in the patterns). This is related to the reason that the memory capacity of a Hopfield network scales linearly with network size. Likewise, memories in larger networks are stored across multiple synapses, so that the network will be more robust to irregularities at single synapses.

## Discussion

Here we have presented the transient attractor network, defined primarily by recurrent excitatory connections that are governed by an associative (Hebbian) plasticity that decays within seconds. We have demonstrated that such a network is capable of a wide range of useful behaviors, including short-term memory (Figs 1–3), source (or stream) segregation (Fig 4), signal de-noising (Fig 5), memory maintenance (Fig 6), top-down modulation (Fig 7). Furthermore, we demonstrated the robustness of the model with respect to both synapse strength and homogeneity (Fig 8). The concept that the same underlying network mechanism might have several uses in sensory computation is compelling in its simplicity. In fact, each of the tasks in Figs 2 and 4–7 was performed using the exact same network with the same parameters. Furthermore, while many of the above functions of transient attractor networks are demonstrated with these simplified networks, the networks size should actually make its desirable properties more robust.

The mechanisms and network structure underlying transient attractors are known to exist in the cortex–except, perhaps, for associative transient plasticity (see below). It does not depend on a set of stable attractors, or some finely prescribed structure. This allows it to be a candidate for short-term memory in a wide variety of regions, such as the primary sensory cortex [14,15]. This is in contrast with a large number of short-term memory models which prescribe such tasks to particularly specialized regions of the brain. The broad applicability of short-term memory benefits from widely applicable mechanisms, perhaps working in tandem with more specialized regions.

### Alternative models for short-term memory

The classic model for short-term memory stores information in persistent attractors [5], that is through a self-sustaining state within the network. Once such an attractor is activated, activity will persist until externally stopped, while the identity of the persistent attractor stores the information. This self-sustenance is typically achieved in neural networks through different combinations of recurrent excitation [4,32], inhibition [33], or both [6,7,34]. Of the many models of persistent attractors, an interesting subset made use of synaptic modifications to the attractor to aid in the persistence of activity [27,35]. The combination of persistent activity and underlying synaptic modifications does resemble the transient attractor network (Fig 3D), but nevertheless information storage in these networks relies on persistent activity. While various experiments [36–40] support the idea of persistent activity underlying short-term memories, a number of conflicting studies in different brain areas have drawn doubt on the universality of such a mechanism [5,11,18].

As a result, other models for short-term memory have been proposed, using processes such as cell assemblies [41], non-stationary activity [42], cross-regional networks [43,44], or purely feed-forward circuits [32]. These other ideas all rely on neural activity for information storage, and thus are still distinct from the idea of storing information in neural connectivity.

Several models have also been proposed which store short-term memories as temporary changes in synaptic strength–as the transient attractor network does–using either direct associative plasticity [17,19,20,22] or synaptic facilitation [18]. In the majority of these, the scope of the memories was pre-defined by the structure of the network. Sandberg et al. [17] used a ring attractor which could store individual variables due to the ring structure, Szatmary and Izhikevich [19] used randomly created periodic attractors, while Mongillo et al. [18] facilitated pre-defined cell assemblies. This is in contrast to the transient attractor network, which considers how recent stimuli might shift the locations of the attractors. In this respect, our model is highly similar to a model recently proposed by Fieberg and Lansner [22], which stored short-term memories in transient associative changes to the connectivity. Our work adds to this idea by demonstrating how such a mechanism occurring within the sensory cortices might assist with a variety of other functions such as temporal coherence analysis, signal denoising, and memory maintenance, combined with analysis of the systems robustness to a variety of perturbations.

### Experimental evidence for transient associative synaptic plasticity

The transient attractor network above relies on an associative learning rule that decays on the order of seconds. There is scattered experimental evidence for transient associative effects (i.e., where strengthening of connectivity occurs between coactive neurons), which has been observed in ferret auditory cortex [29], macaque ITC [45], and dissociated networks [46]. It is known that associative learning takes place over a variety of timescales due to multiple mechanisms [47], including some direct associative connections which decay in minutes [48,49]. It is conceivable such processes might exist for shorter timescales, but have proven difficult to separate from non-associative plasticity similar timescales (such as synaptic facilitation and depression). Such associative plasticity also may be possible to achieve associative changes in effective coupling using non-associative facilitation within certain network structures; this is the subject of future work.

### Extensions of the transient attractor network

It is hypothesized that the pre-existing wiring of neural networks in sensory cortices is informed by the structure of natural stimuli [50], which is equivalent to non-uniform connectivity (*S*_*ij*_) in the transient attractor network. While such non-uniformity would bias the network towards some attractors, this could be advantageous in sensory cortex, as the location of transient attractors will be guided both by the immediate history and by the pre-learned nature of typical stimuli. When presented with a novel stimulus, the network’s interpretation may be biased by learned stimuli, which are presumably the stimuli that have proven the most useful (given rules of long-term plasticity). This coordination of short- and long-term plasticity is distinct from earlier work that stored short-term memories by strengthening some pre-existing attractors: in the transient attractor model, recent activity may change the nature of (e.g. strengthen, make stable or shift) pre-existing attractors. This allows for much greater flexibility in memory storage; the number of possible transient attractors (as influenced by pre-learned patterns, recent history, and by the nature of the instantaneous input) is far larger than that of pre-existing attractors.

## Methods

### Neuron model

In our model, the firing rate of neuron *i* at time *t, y*_*i*_(*t*) is governed by the neuron’s instantaneous membrane potential, *v*_*i*_(*t*). The dependence of firing rate on the potential is described using a saturating, rectified linear function

$$y_{i}(t)=\max\left[1-\exp(a(b-v_{i}(t))),0\right]$$ (4)

The membrane potential evolves proportional to the sum of the recurrent excitatory *I*_*Exc*_(*t*), inhibitory *I*_*Inh*_(*t*), input *I*_*in*_(*t*) and leak *I*_*Leak*_(*t*) currents,

$$\frac{dv_{i}(t)}{dt}=\frac{1}{\tau_{Leak}}(I_{Exc}(t)+I_{Inh}(t)+I_{In}(t){+I}_{Leak}(t))$$ (5)

$$I_{Exc}(t)=\sum_{{Exc}_{j}}W_{ij}(t)y_{j}(t)$$ (6)

$$I_{Inh}(t)=W_{Inh}y_{Inh}(t)({E_{rev}^{I}-v}_{i}(t))$$ (7)

$$I_{Leak}(t)=-v_{i}(t)$$ (8)

Note that the excitatory and inhibitory recurrent currents are themselves a weighted sum of other neurons’ firing rates (with weight *W*_*ij*_(*t*) between excitatory neuron *i* and *j*, and *W*_*Inh*_ from the inhibitory neuron to all excitatory neurons). Finally, the inhibitory current acts to return the membrane potential to the inhibitory reversal potentials ($E_{rev}^{I}$), while the excitatory currents are independent of the membrane potential; this is simplification is valid since the excitatory reversal potential is far larger than typical values for the membrane potential, so that the difference between the two is approximately constant.

### Parameters

Simulation parameters which remain constant across all simulations are listed in Table 1. Weights between neurons depend on the network structures used in each Figure, as follows:

For Fig 1: *W*_*SE*_ = 5, *W*_*IE*_ = 5, *W*_*II*_ = 20, *W*_*EI*_ = 10, *W*_*EE*_ = 1

For Fig 3 (ring attractor): *W*_*SE*_ = 1, *W*_*IE*_ = 10, *W*_*EI*_ = 2, *W*_*EE*_ = 1.5

For Figs 2 and 4–8: *W*_*SE*_ = 5, *W*_*IE*_ = 5, *W*_*II*_ = 20, *W*_*EI*_ = 1, *W*_*EE*_ = 0.1

**Table 1:** Simulation parameters.

Name	Symbol	Value
Max Hebbian	*H*_*max*_	5
Min Hebbian	*H*_*min*_	1
Facilitation increase	$\tau_{H_{+}}$	100ms
Facilitation decrease	$\tau_{H_{-}}$	200ms
Depression increase	$\tau_{x_{+}}$	50ms
Depression decrease	$\tau_{x_{-}}$	100ms
Leak current	*τ*_*Leak*_	1ms
Firing scale	*a*	1
Firing threshold	*b*	1

Ring model (Fig 3): The profile of the recurrent excitatory baseline weights across space follow a Gaussian bell curve with a standard deviation of 10 centered at the postsynaptic neuron’s location, and a strength of 1.5 in the center (recorded in Parameters above). All weights are then multiplied by a random noise term, drawn from normal distribution, μ = 1, σ = 0.05.

Temporal Coherence Model (Fig 5): Time courses were generated using a continuous low-pass filter applied to Gaussian noise; in particular, a filter was used in which the energy at a frequency *f* was multiplied by exp(-0.1*f).

De-noising model (Fig 6): The signal pattern was deliberately chosen for its distinctive shape; the pattern was then used to classify all input channels as either signal or non-signal. The signal channels were only ever active when a significant number of the other signal channels were active. In particular, an occluded pattern (a subset of 75% of all signal channels) was shown for the initial 25 ms of each 100 ms window. The subset included was chosen in a manner that meant the occluded pattern would be spatially continuous. In contrast, the activity of each non-signal channel was composed of 25 ms long bursts of activity. At any time, each dormant non-signal channel had a constant probability of starting a burst. This probability was selected so that the average activity across non-signal channels is equal to average activity in signal channels.

Robustness analysis (Fig 8): In order to test robustness to changes in synaptic strengths, the baseline strength for each type of connection was modified until the memory recall is no longer ‘successful’. The change in baseline strength was applied to all connections of any single type, and the default case used was that presented in Fig 2. Recall was deemed ‘successful’ if, during relevant probe, the average firing rate within either pattern was at least 0.1 (10% of the maximal firing rate), and at least five times greater than the average firing rate of the most active non-pattern channel.

The sensitivity to sparsity was tested by changing the density of recurrent connections. 20 different sparsity values were tested (from 0.05 up to 1, with a step size of 0.05), with 100 trials at each value. The recurrent connection matrix was then randomly set using according to the sparsity value; each connection was independently set to zero with probability = 1 –density. All the remaining weights were then scaled uniformly to ensure that the total strength of recurrent excitatory connections remained constant. For each trial, two random patterns were selected, with each pattern being a subset of 20 randomly selected excitatory neurons. From each of these patterns a probe (a subset of 5 neurons) was then selected. The results record the behavior of the various neurons after training in the presence of the probe; because the probe neurons are externally stimulated, they were excluded from the analysis. Each excitatory neuron was considered active if its average firing rate was over 0.1 while the probe displayed. These results were then summarized using two measures. The first of these, Positive Predictive Value (PPV). This represents what proportion of cells that were active were actually members of the appropriate pattern (that is, the pattern which matches the probe used). The second measure used is the True Positive Rate (TPR), which is the proportion of the neurons from the appropriate pattern which were active. These two measures combined give a complete description of how the different populations of neurons reacted to the probe.

### Source code

All code was written in MATLAB, and is accessible as supplementary information (S1 File).

## Supporting information

S1 File MATLAB code for all simulations. (ZIP)
